# A computational approach identifies two regions of Hepatitis C Virus E1 protein as interacting domains involved in viral fusion process

**DOI:** 10.1186/1472-6807-9-48

**Published:** 2009-07-29

**Authors:** Roberto Bruni, Angela Costantino, Elena Tritarelli, Cinzia Marcantonio, Massimo Ciccozzi, Maria Rapicetta, Gamal El Sawaf, Alessandro Giuliani, Anna Rita Ciccaglione

**Affiliations:** 1 Department of Infectious, Parasitic and Immune-mediated Diseases, Istituto Superiore di Sanità, Rome, Italy; 2 Medical Research Institute, Alexandria University, Alexandria, Egypt; 3 Department of Environment and Primary Prevention, Istituto Superiore di Sanità, Rome, Italy

## Abstract

**Background:**

The E1 protein of Hepatitis C Virus (HCV) can be dissected into two distinct hydrophobic regions: a central domain containing an hypothetical fusion peptide (FP), and a C-terminal domain (CT) comprising two segments, a pre-anchor and a trans-membrane (TM) region. In the currently accepted model of the viral fusion process, the FP and the TM regions are considered to be closely juxtaposed in the post-fusion structure and their physical interaction cannot be excluded. In the present study, we took advantage of the natural sequence variability present among HCV strains to test, by purely sequence-based computational tools, the hypothesis that in this virus the fusion process involves the physical interaction of the FP and CT regions of E1.

**Results:**

Two computational approaches were applied. The first one is based on the co-evolution paradigm of interacting peptides and consequently on the correlation between the distance matrices generated by the sequence alignment method applied to FP and CT primary structures, respectively. In spite of the relatively low random genetic drift between genotypes, co-evolution analysis of sequences from five HCV genotypes revealed a greater correlation between the FP and CT domains than respect to a control HCV sequence from Core protein, so giving a clear, albeit still inconclusive, support to the physical interaction hypothesis.

The second approach relies upon a non-linear signal analysis method widely used in protein science called Recurrence Quantification Analysis (RQA). This method allows for a direct comparison of domains for the presence of common hydrophobicity patterns, on which the physical interaction is based upon. RQA greatly strengthened the reliability of the hypothesis by the scoring of a lot of cross-recurrences between FP and CT peptides hydrophobicity patterning largely outnumbering chance expectations and pointing to putative interaction sites. Intriguingly, mutations in the CT region of E1, reducing the fusion process *in vitro*, strongly reduced the amount of cross-recurrence further supporting interaction between this region and FP.

**Conclusion:**

Our results support a fusion model for HCV in which the FP and the C-terminal region of E1 are juxtaposed and interact in the post-fusion structure. These findings have general implications for viruses, as any visualization of the post-fusion FP-TM complex has been precluded by the impossibility to obtain crystallised viral fusion proteins containing the trans-membrane region. This limitation gives to sequence based modelling efforts a crucial role in the sketching of a molecular interpretation of the fusion process. Moreover, our data also have a more general relevance for cell biology as the mechanism of intracellular fusion showed remarkable similarities with viral fusion

## Background

Hepatitis C virus (HCV) is a positive-strand RNA virus that belongs to the family of *Flaviviridae *[1]. The genome of HCV encodes for two envelope glycoproteins designated as E1 and E2 respectively. E1 and E2 are classified as type I transmembrane (TM) glycoproteins, and show a N-terminal ectodomain and a C-terminal TM domain. The El and E2 proteins interact to form a noncovalent heterodimer which is present at the surface of the viral particle and mediates the entry of HCV into host cell [2]. After viral binding to a cellular receptor(s) and endocytosis, the E1E2 complex is thought to induce fusion between the viral envelope and a membrane of an internal compartment of host cell [3,4]. However, the definite identification of the fusion protein is still lacking.

A common property of the fusion proteins of other members of the *Flaviviridae *family, such as tick-borne encephalitis virus (TBE) and dengue virus, is their presence at the viral surface as a dimer that, when activated by an appropriate trigger (acidic pH in the endosome), undergoes a transition to a trimeric state. These structural rearrangements expose a hydrophobic domain, called fusion peptide or fusion loop, allowing its insertion into the host cell membrane [5].

Fusion proteins have been divided into class I (Retroviruses, Orthomyxoviruses) and class II (Flaviviruses, Alphaviruses) proteins on the basis of their different structure. However, similarities in the post-fusion conformation suggest that the corresponding fusion processes are mechanistically related [5-8]. In the pre-fusion conformation the TM and FP segments are at the opposite ends of the fusion protein: the TM is anchored to the viral membrane while the FP is inserted into the host cell membrane. In the following steps, the protein folds back on itself directing the C-terminal TM anchor towards the fusion peptide along with their associated membranes. These structural changes lead to a final highly stable rod-like conformation in which the TM and the FP domains are at the same end of the molecule and are closely juxtaposed in the same fused membrane [5]. This transition suggests the physical interaction between the TM anchor region and the fusion peptide as one of the key events that force the cellular and the viral membranes into close apposition to trigger a complete fusion.

The similarities with other Flaviviruses suggest that HCV may harbour a class II fusion protein, however its identification is still controversial. Recent data suggest that both E1 and E2 may participate in the membrane fusion by a complex mechanism which involves multiple hydrophobic regions of these glycoproteins [4,9]. Although the specific function of each region is still not defined, it has been proposed by several groups that the E1 protein could play an active role in the fusion process as it contains the putative fusion peptide of HCV [9-14].

**Figure 1:**
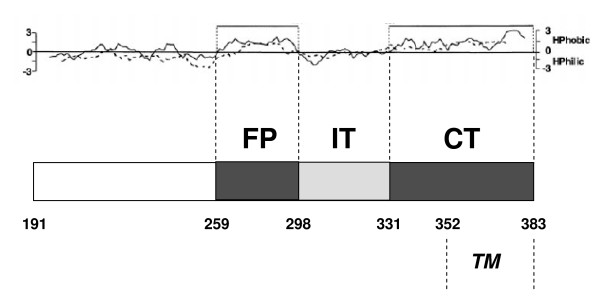
**Schematic diagram of the E1 regions analyzed in this study**. The Kyte & Doolittle (continuous line) and Goldman (dashed line) hydrophobicity plots of the E1 protein is shown at the top. The studied E1 fragments are indicated as shaded boxes. The trans-membrane (TM) domain is also shown. Key amino acid positions are indicated.

The analysis of the secondary structure of E1 reveals that it contains two long hydrophobic regions which show membrane-associating properties [10,15] and are strongly conserved among all HCV isolates [16]. One region is localized in a central position (aa 259–298) and the other (aa 331–383) at the C-terminal end (reported as FP and CT, respectively, in Fig. 1). The central segment has been suggested to contain the putative fusion peptide of HCV [9-14]. The hydrophobic CT region contains an amphipathic pre-anchor domain (aa 331–347) [17] followed by the TM region (aa 353–383) [18]. Recently, it was shown that a fragment of E1 (aa 317–339), which includes part of the hydrophobic region of pre-anchor, is capable of destabilizing model membranes [14,19]. Interestingly, HCV pseudoparticles (HCVpp) containing mutations in each of the hydrophobic domains of E1 showed reduced fusion property [9,11,20].

In the present paper, we used a computational approach to evaluate the possible interaction between the hydrophobic regions of E1, FP and CT (Fig. 1), as a potential key step in the mechanism of membrane fusion. To model this interaction, we analysed a dataset containing sequences from 5 HCV genotypes by means of two independent computational approaches. The first one is a sequence alignment method building upon the hypothesis that two interacting sequences, given the need to satisfy shared physical interaction constraints, display a much greater degree of co-evolution (estimated in terms of correlation of sequence variability among different strains) than two non-interacting proteins. This paradigm was demonstrated to be very effective in detection of interacting peptides [21,22].

The second one was based upon Recurrence Quantification Analysis (RQA) [23] as applied to the putatively interacting sequences transformed into numerical series by the coding of residues with their relative hydrophobicity [24]. After this step, the amount of different size patches of the two putative partners displaying a similar hydrophobic patterning is scored so to derive some global measures of hydrophobic patterning cross-correlation between the two partners. This method is based upon the large body of evidence indicating two mutually binding regions tend to share the same hydrophobic patterning so both minimizing the energy of the complex (thermodynamic constraint) and maximizing the amount of time the two partners stay aside, so increasing the interaction probability (kinetic constraint) [25,26]. RQA in turn was demonstrated extremely efficient to predict protein-protein interaction in different settings [24,27-30].

## Results

### Analysis of E1 domains by sequence alignment/co-evolution method

To evaluate the probability of the hypothesized interaction between the two hydrophobic regions of E1 protein (Fig. 1, see FP and CT), we assembled a sequence dataset by retrieving from public database twelve E1 protein sequences belonging to 5 different HCV genotypes and, in the case of genotype 1, two subtypes (1a, 1b, 2a, 3a, 4a, 5a) (see Methods for accession numbers). A fragment from the Core protein sequence (aa 39–74), simply called "Core" for the sake of brevity in the present paper, was selected from a conserved region of the Core protein showing high content of charged amino acids (R and K); this region appears a good control for our analysis, because it may represent a HCV-RNA binding site and, thus, it is unlikely to be an interacting site for E1 in the viral particle [31].

The dataset was analyzed by the co-evolution method based on the comparison between the inter-strains sequence superposition computed on FP and CT peptides, respectively. This analysis is based on the observation that two interacting proteins have a much more "constrained" evolutionary space than a pair of non interacting proteins and consequently much less room for random genetic drift [21,22]; this ends up into a much higher resemblance of the between strains differences measured over interacting proteins than over random protein pairs. On this basis, we compared the correlation between the distance matrices relative to HCV genotypes for FP and CT domains (autologous comparison, i.e. comparison inside the same E1 protein) with the correlation of the distance matrices computed over both FP and CT peptides with the distance matrices computed over a fragment of the HCV Core protein (heterologous comparison, i.e. comparison with an independent protein). It is important to stress that we are not comparing the relative amount of sequence homology between the different peptides, but simply if the "between genotypes" similarities for the same peptide remain more invariant in FP-CT comparisons than in FP-Core and CT-Core comparisons.

Analysis was carried out by applying ClustalW to all the possible genotype pairs separately for the different peptides so to generate three distinct "between genotypes" distance matrices, one for each peptide (FP, CT and Core). Then the three distance matrices were each other correlated giving rise to the following mutual correlations: FP/CT = 0.62, FP/Core = 0.43 and CT/Core = 0.44. These values are indicative of a greater correlation between FP and CT, according to their supposed interaction. However, the evidences are still preliminary being the cross-correlation between interacting pairs only marginally different from the non-interacting pairs. This probably comes from the relatively low random genetic drift between HCV genotypes that is a feature frequently observed in viruses [27]. This low random genetic drift generates an artificially high correlation even for non interacting pairs.

### Analysis of E1 domains by RQA technique

To overcome the limitations of the sequence homology approach, we then applied the Recurrence Quantification Analysis (RQA) approach [27]. This method investigates the presence of cross-correlations between different hydrophobicity patches of the analysed peptides, so to highlight the possible presence of hydrophobic zippering interaction motifs [30,32].

In order to have a quantitative appreciation of the cross recurrences we used two numerical descriptors of the amount of cross recurrence: the percentage of recurrent pairs, called REC, and the percentage of "deterministic recurrences", called DET [23,24] (see Methods for details).

**Figure 2:**
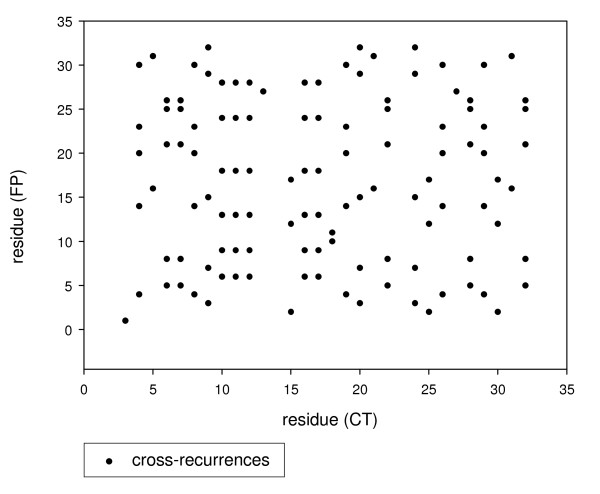
**An example of cross-recurrence plot of the FP-CT comparison (genotype 1b)**. Each black dot represents a cross-recurrence, i.e. very similar hydrophobicity between the compared amino acid residues from FP and CT peptides.

As shown in Fig. 2, the cross-recurrence plot for FP and CT peptides of genotype 1b revealed that the two peptides display a lot of cross-recurrences (patches with very similar hydrophobicity patterns) that could mark hydrophobic regions of mutual interaction; moreover these regions appear in ordered columns so stressing the plausibility of spots of mutual interaction between peptides. The different length of FP and CT peptides (30 and 53 residues respectively) constrained us to work on contiguous (shifted by one residue at time) versions of the CT peptide so to match the FP peptide with subsequent equal length (30 residues) peptides. These subsequent windows give rise to almost identical cross-recurrence values for a given genotype, so we can safely substitute the average cross-recurrence parameters for each comparison as specific genotype cross-recurrence values (REC and DET).

In addition to the FP and CT comparison, we included as control sequence another fragment of the E1 protein (aa 299–330), called IT in the present paper (Fig. 1). This fragment is highly conserved in HCV [31] and contains an epitope for human neutralizing monoclonal antibodies, suggesting its location on viral surface [33]. For this reason, it is not expected to participate directly to the physical interaction between the FP and CT portions into the membrane but nevertheless is expected to display a greater resemblance with both FP and CT with respect to Core for its pertaining to the same integrated system (E1 protein).

As a matter of fact, being IT a fragment of E1 protein as FP and CT, it cannot be absolutely excluded that even the IT sequence follows the co-evolution drive of FP and CT, thus, we included a further control fragment from a completely different HCV protein, i.e. Core protein. The Core fragment, previously described; was shown to interact with viral RNA and, thus, supposed to be located inside the virion capsid, in a completely different location with respect to both FP and CT and thus it is not expected to co-evolve with these latter fragments of E1.

The strategy to include the described controls (IT and Core) allowed to check two effects: (a) the inside-protein and (b) the autologous/heterologous relations. In fact, the need of having a global optimised protein structure could insert some cross-correlation among amino acid patches of the same protein, even if they do not physically interact. If the hypothesized model is correct we thus expect a descending order cross correlation such as: CT-FP > CT-IT > CT-Core.

**Figure 3:**
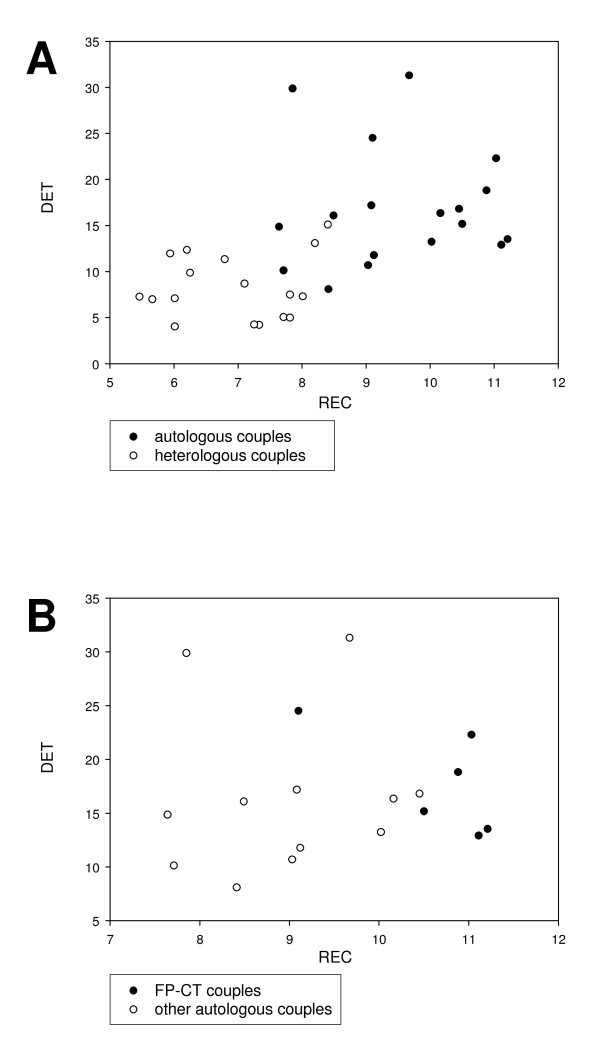
**DET and REC values from (A) autologous and heterologous couples and from (B) FP-CT and other autologous couples**. (A) Autologous pairs (FP/IT, FP/CT, IT/CT) (black dots) are more correlated than heterologous ones (FP/Core, CT/Core, IT/Core) (white dots). (B) Differently from other autologous couples (white dots), FP/CT couples (black dots) seem to occupy (with one only exception) the most extreme right portion of the graph, so indicating a possible preferential FP-CT attachment pairs.

In Fig. 3A, the different couples of peptides are sampled in the REC/DET plane with autologous (FP/IT, FP/CT, IT/CT) and heterologous pairs (FP/Core, CT/Core, IT/Core) reported as black and white dots, respectively. The difference between the two conditions is striking: the amount of cross correlation indicated that autologous pairs were more correlated than heterologous (FP/IT, FP/CT, IT/CT > FP/Core, CT/Core, IT/Core).

In order to get a statistical significance appreciation of this difference we performed an analysis of variance on both REC and DET descriptors taking into consideration both the autologous/heterologous difference and the eventual differences between genotypes (some genotypes having a greater cross-correlation than others). To obtain a more reliable and comprehensive score of the entity of cross-correlation between different peptides we took advantage of the correlation existing between REC and DET (Pearson r = 0.53, p < 0.001). The existence of such a correlation allowed to compute a first principal component of the REC/DET plane positively correlated with REC and DET (r = 0.87 with both REC and DET) and explaining 76% of the total variability of the bi-dimensional REC-DET space [see Additional file 1]. This component (Factor 1) can be considered as a cumulative score collecting the common portion of variation of the two REC and DET indices, thus allowing for a more robust estimate of the actual cross correlation of the different couples as for hydrophobicity patterning reminiscent of the physical interaction between the corresponding peptides. The general dataset after computation of the first principal component is shown in a supplementary file [see Additional file 2].

In order to get the statistical significance of the autologous/heterologous comparison an Analysis of Variance was carried out. The dependent variables were REC, DET and Factor1 scores, and the independent sources of variation were the type of relation (autologous/heterologous) and the genotype. In this way, together with the autologous/heterologous effect, we could check if a significant "among-genotypes" difference was present. Results of this analysis are reported in a supplementary file [see Additional file 3].

All the three descriptors were highly statistically significant in the autologous/heterologous comparison, so pointing to a neat "protein effect" in maintaining a general shape of the protein. Factor1 was demonstrated to be the most significant index (Rsquare = 0.74): this was expected, as such a descriptor retains only the correlated information of both REC and DET. REC was more efficient than DET in discriminating the two classes, while no significant genotype effect was singled out, indicating the observed effect is common to all the genotypes and thus indirectly indicating, as expected, the same interaction occurs in all genotypes. If we consider a given protein as a global ensemble with non-local rules shaping its conformational space, this is a very important result. In some sense, it is like the entire sequence presents the need of a 'self-interaction' between different portion of the molecule driven by their mutual similarity [26,34]. We can think the FP-IT-CT system as shaped, in terms of hydrophobicity patterning, by the need of carrying out a physical interaction. This need is clearly not present between the Core fragment and the FP-IT-CT peptides, as shown by a clear decrease in hydrophobic patterning correlation of the heterologous couples. The descriptive statistics of these indices is reported in Table 1.

**Table 1:** Descriptive statistics of REC, DET and Factor1 indices in autologous and heterologous comparison

		Autologous				Heterologous			
Variable	N	Mean	Std Dev	Min	Max	Mean	Std Dev	Min	Max

REC	18	9.525	1.205	7.640	11.210	6.947	0.935	5.460	8.400

DET	18	16.876	6.434	8.100	31.310	8.330	3.342	4.050	15.120

Factor 1	18	0.804	0.696	-0.391	2.093	-0.804	0.452	-1.489	0.272

If we focus only on the comparison among the autologous pairs of peptides (Fig. 3B), the FP/CT couples (black dots) seem to occupy (with one only exception) the most extreme right portion of the graph, so indicating a possible preferential FP-CT attachment pairs. To give a statistical basis to this observation a Student's t-test was performed (Table 2). All the three descriptors showed highest average scores in FP/CT pairs with respect to "other" couples, REC, DET and Factor1 all reached the statistical significance with an expected higher sensitivity for Factor1 (that by construction is filtered out of noisy information) (p < 0.003 and p < 0.04 for REC and DET respectively and p < 0.0001 for Factor1). Thus, we can safely state that the amount of cross recurrence of the FP/CT couples is significantly higher with respect to FP/IT and CT/IT, so pointing to the existence of a some sort of FP/CT interaction. This result is the proof of the FP-CT > (CT-IT and FP-IT) > (CT-Core and FP-Core) order of cross-correlation we hypothesized in our model.

**Table 2:** Statistic description of REC, DET and Factor 1 indices in autologous comparisons.

	Couple = other (FP/IT, CT/IT)				Couple = FP/CT				
Variable	N	Mean	Std Dev	Min	N	Mean	Std Dev	Min	Max

REC	12	8.969	0.972	7.640	6	10.638	0.793	9.100	11.210

DET	12	16.374	7.258	8.100	6	17.880	4.801	12.920	24.520

Factor 1	12	0.572	0.728	-0.391	6	1.268	0.307	0.989	1.780

Statistical significance of observed REC, DET and Factor 1 values in the two groups by Student's t-test: REC p < 0.003; DET p < 0.04; Factor 1 p < 0.0001.

### RQA analysis of FP-CT interaction as compared with NS4B-CT and E1TM-E2TM

In order to go in depth into the proof of the suggested model, we evaluated the possibility that the evidence of a strongest interaction of the FP-CT pair with respect to both CT-Core and FP-Core ones could be driven by the fact we are correlating two membrane peptides in the FP-CT case. In fact, membrane location of a peptide imposes a deterministic structuring to hydrophobicity patterning that could be at the basis of the observed FP-CT cross recurrence structure.

In order to operate a more stringent control of the FP-CT interaction, the amino-terminal fragment (aa 1712–1737) from the NS4B protein of HCV was used as further negative control for RQA. Indeed, this fragment was previously reported to interact with membranes [35] and, being NS4B a non-structural protein, was not expected to interact with the FP-CT system. The NS4B fragment will be simply named "NS4B" further in the text.

The graphical comparisons in terms of determinism (DET) of the FP-CT with both NS4B-CT (upper panel) and Core-CT (lower panel) negative control pairings are reported in supplementary file [see Additional file 4]. In both cases FP-CT couples show a greater DET than the negative controls (with the only exception of genotype 1b that in any case is very near to the identity line) so pointing to the relevance of FP-CT interaction not merely determined by "membrane location".

To obtain a more clear assessment of the proposed interaction model, we included in the analysis a positive control. The trans-membrane C-terminal end (aa 717–745) of E2 protein was experimentally demonstrated to have physical interaction with the trans-membrane C-terminal end (aa 352–383) of E1 [20]. Thus, we subjected these fragments (named E2TM and E1TM, respectively) to RQA. The behavior of E1TM-E2TM interaction is reported in a supplementary file [see Additional file 5]. Here the significance of the results are still more clear: the positive control has consistently (and to a large extent) higher DET values than both NS4B-CT (upper panel) and Core-CT (lower panel) negative control pairs.

As last point we compared the FP-CT pair and the E1TM-E2TM positive control in both the REC and DET spaces. Although E1TM-E2TM interaction is consistently predicted as stronger in the DET plane (upper panel), this is not the case in the REC plane (lower panel) where the two interactions are more or less equivalent (sample points disposed on both sides of the identity line) [see Additional file 6], further supporting the plausibility of our FP-CT interaction model.

A very important additional proof of the proposed model is the check of the "sequence order dependence" of the obtained results, carried out by random scrambling the amino acid order of the analyzed fragments. In the RQA only DET parameter is affected by amino acid order (REC parameter is only dependent on amino acid composition). Thus, if the observed interaction strength between two peptides in terms of the DET descriptor remains unchanged after random scrambling, this points to a largely unspecific interaction between the two elements of the pairs. In contrast, if the random shuffling of residue order determines a marked drop of the DET variable, this implies the native arrangement of the residues is crucially important for driving the interaction. In a supplementary file [see Additional file 7] it is shown that both E1TM-E2TM and FP-CT interactions were destroyed by randomly scrambling the residue order, with the "native" sequences (position indicated by an arrow) having a greater DET value than any scrambled sequence over a set of twenty (positions indicated by black dots). As expected, NS4B-CT interaction (negative control in RQA analysis) had a DET value similar to the shuffled copies, so giving a further confirmation to the FP-CT interaction.

Having obtained a multifaceted proof of the plausibility of the FP-CT interaction, we carried out a finer analysis by studying the effect of mutations on this interaction system.

### RQA analysis of E1 fusion mutants

In the last years, E1 mutations affecting viral fusion of HCV 1a pseudotyped particles *in vitro *have been described [9,11,20]. To evaluate whether hydrophobicity patterning correlation could be influenced by such mutations, we inserted them in the FP and CT domains of the same genotype 1a, H77c isolate [GenBank:AF011751], and analysed the resulting sequences by RQA. Only the mutations for which a frank decrease in biological effect was observed were selected for the analysis (see Table 3). Some simulated double mutations for which we had no biological data were also computed in order to check the relative sensitivity of cross-correlation parameters to FP and CT patches.

**Table 3:** Mutations in E1 protein affecting viral fusion *in vitro*

E1 domain	aa substitution	References
FP	Y276F	(Lavillette et al., 2007)
	G282D	(Lavillette et al., 2007)
	Y276R	(Drummer et al., 2007)
	Y276A	(Drummer et al., 2007)
	F285A	(Drummer et al., 2007)
		

CT	V333A	(Drummer et al., 2007)
	L337A	(Drummer et al., 2007)
	M347A	(Drummer et al., 2007)
	L356W	(Ciczora et al., 2007)
	A360W	(Ciczora et al., 2007)

If cross RQA is a sensitive indicator of the interaction strength we expect a diminished cross-recurrence between hydrophobicity patterning of the mutant peptides. It is worth noting this is a much harder test than the one described before. While the above analysis was based upon the differences in cross-correlation between interacting and non-interacting pairs, here we try and model the different relative strength of interaction inside an interacting pairs, i.e. we are dealing with interaction modulation and not simply with its presence/absence. The observed cross-recurrence between different FP/CT pairs was computed by means of REC and DET descriptors together with another typical RQA descriptor called laminarity (LAM) that corresponds to the percentage of vertical (horizontal) recurrent lines in the cross recurrence plot. The addition of this descriptor to REC and DET was dictated by both the need of modelling a very subtle effect (indeed the different couples differ among them for only one residue out of 40) and by the fact that interaction is a strict order dependent process in which the spatial disposition of cross recurrences (as measured by both DET and LAM) is much more crucial than their actual number (as measured by REC). Table 4 reports the results obtained by the application of cross recurrence to the different peptide couples. As expected REC was strongly invariant among all the couples; in contrast both DET and LAM (strongly order dependent parameters) show a drastic decrease in double mutants and in all the CT mutants. The decreases we observed in both DET and LAM descriptors are approximately one half the wild type respective value. This is a very remarkable result if we consider it is due to one residue only substitution. No fusion mutant couple showed a significantly increased cross correlation with respect to wild type and mutation of the sole FP did not produce significant changes in cross-correlation. The above results suggest that mutations on CT patch are in general more sensitive in terms of alteration of hydrophobicity pattern (the double mutants have variation with respect to wild type comparable to what observed with CT mutations only). The lack of sensitivity of the method as for only FP mutations probably points to a diminished strength of hydrophobic patterning interaction constraints for FP peptide with respect to CT.

**Table 4:** RQA analysis of different wild type (wt) or mutant FP and CT peptide couples

Sequence type	FP	CT	REC	DET	LAM
wild type	wt	wt	13.00	26.32	24.81

FP mutant	Y276R	wt	12.90	26.50	25.00

FP mutant	Y276A	wt	13.50	27.50	23.80

FP mutant	Y276F	wt	13.10	27.60	26.60

FP mutant	G282D	wt	12.89	29.50	25.00

FP mutant	F285A	wt	13.10	27.15	25.20

CT mutant	wt	V333A	11.72	10.83	13.33

CT mutant	wt	L337A	11.52	10.17	10.17

CT mutant	wt	M347A	12.01	13.82	21.13

CT mutant	wt	L356W	11.72	16.67	21.67

CT mutant	wt	A360W	11.84	15.26	20.81

double mutant	F285A	V333A	12.60	17.82	12.40

double mutant	Y276R	L337A	11.40	11.97	10.26

double mutant	Y276A	M347A	12.70	17.70	20.00

*: the amino acid change of mutated versions is reported

All in all, the mutation analysis gives further strength to the direct physical interaction hypothesis of FP and CT peptides.

## Discussion

In this report we have presented evidences indicating that the C-terminal hydrophobic region of HCV E1 protein can interact with the inner putative fusion peptide. This physical interaction is likely at the basis of the strong cross-correlation between the hydrophobicity patterning of these two domains and it may be part of a mechanism of protein refolding which leads to viral fusion to intracellular membranes.

Our analysis was performed at a first level by the scoring of an higher correlation in the "between strains" distance matrices relative to the homologous (FP/CT) *vs*. the heterologous (FP/Core, CT/Core) pairs. This gave us the idea that some constraints to the random genetic drift were present between homologous couples that were absent in heterologous ones. The second level of analysis was reached by the demonstration of a meaningful correspondence between hydrophobicity patterning of homologous couples and that this correspondence was higher for fusion peptide and C-terminal region pairs. This allows us, given the crucial role of hydrophobicity for both folding and protein protein interaction, to infer a physical interaction between the two peptides. Moreover, if variations of the entity of correlation between the two peptide patterning are able to discriminate different HCVpp fusion mutants, we can safely affirm that not only the two peptides interact in some way but that their effective interaction can be quantitatively modeled on the basis of their sequence. This may open the way, on the long run, to rationally designed peptide based therapy [25]. The fact FP mutations are not detectable by this method has two-fold implications. First, hydrophobicity is not the sole driving force of interaction process and other elements like topology, steric hindrance, flexibility are important actors of the play: the 'FP side' of interaction could be more sensible to these other factors. Second, it is conceivable that the described mutations interfere with the first step of viral fusion. Indeed, they might block the insertion of the fusion peptide into cell membranes without affecting the following interaction with the C-terminal anchor.

Although a fusion peptide has not been defined for E2, three regions of this protein (aa 418–432, 597–620 and 675–699) [9,36] as well as its TM domain [20] have been proposed to play a role in the fusion process by a still unknown mechanism. The apparent lack of a fusion peptide in E2 supports the hypothesis that this protein might be indirectly involved in the fusion process by controlling the conformational changes of E1E2 complex. Our data seem to favour a view in which the fusion peptide and the C-terminal anchor region of E1 play an active and direct role in the fusion process of HCV, contributing to the first step which leads to hemifusion of viral and cell membranes, while other segments of E1 and E2 may contribute to subsequent pore enlargement via major structural changes. Recently it has been proposed an alternative mechanism that involves the cooperation of the fusion peptide of E1 with a putative stem region (aa 675–699) of E2 [11]. This hypothesis is based on mutagenesis studies which indicated that this last region is involved in E1E2 heterodimerization and in viral entry [36], two typical functions of the Flavivirus stem domain. However, an important function of the stem is also to bring the TM anchor of the protein towards the fusion peptide of the same molecule. This is an universal property of all viral fusion proteins, common to both class I and class II molecules; thus, the cooperation of two different proteins (i.e. E1 and E2) during this step of membrane fusion, though possible, appears unlikely, as it would represent a novel mechanism never described for any virus.

Viral fusion proteins of class II are elongated molecules composed almost entirely of β strands and contain three domains: the centrally located domain I; domain II which is located at one side of domain I and contains the fusion loop at its tip; and domain III which is connected at the other side of domain I. Domain III is also connected by a region called stem or pre-anchor, (50 aa) in E protein of TBE, to the TM domain of the protein. Proteome computational analyses indicate that HCV E1 seems to be missing much of the stem region relative to the E protein of TBE and represents the minimal class II fusion protein structure required to mediate the virus-cell fusion [13]. As for the stem region of E protein of TBE, the HCV E1 pre-anchor sequence was also predicted to be an amphipathic α helix with characteristics of a leucine zipper [13,17]. Moreover, mutagenesis of single positions in this region affects viral fusion [11]. Finally, it was shown that two synthetic peptides of E1, containing part of the hydrophobic region of pre-anchor, showed fusion activity on liposome membrane (aa 317–339) as well as the ability to destabilize model membranes (aa 309–340), a distinctive feature of fragments involved in membrane fusion [14,19].

Interestingly, in the class II fusion model the role of the stem region would be to pull the TM anchor towards the fusion loop and to tightly interact with sites located in domain II in the final post-fusion conformation. It has been proposed that the binding of the stem forces juxtaposition of the fusion peptide loop and the TM segment, driving the opening of an initial fusion pore [6]. Most importantly these functions may suggest strategies for inhibiting Flavivirus entry. Indeed, peptides derived from stem sequences could block completion of the conformational changes by interfering with stem binding sites. Our analysis indicates that amino acids substitutions in the pre-anchor region of E1 (V333A, L337A and M347A) strongly reduced the amount of cross-recurrence suggesting a possible interaction between this region and the domain containing the fusion peptide. Interestingly, a similar association of the trans-membrane domain with the fusion peptide seems to represent a key event in the fusion process of influenza virus [37]. If the interaction between these two domains of E1 is required to complete the fusion process, this step could be a potential target for antiviral fusion inhibitors. An analogous strategy has been used to inhibit HIV-1 entry, in this case peptides corresponding to the C-terminal ectodomain of the gp41 fusion protein bind to the viral protein and block HIV-1 infection by preventing its conformational changes [38]. This approach was exemplified by the HIV-1 peptide T20/Enfuvirtide, a licensed antiretroviral drug, which was developed to interfere with gp41 protein refolding to the final post-fusion structure [39,40].

## Conclusion

Our findings suggest a physical interaction of the putative fusion peptide and the C-terminal region of HCV E1 protein. This view is supported by (1) data from sequence homology analysis, (2) high level of cross-recurrence between the two domains (3) the sequence dependence of the observed interaction strength and (4) the destroying effect of single mutations in the C-terminal region on the cross-recurrence. Consistent with their reported membrane-active properties, interaction of these peptides implicates they could be key players in the fusion process.

Though carried out on HCV sequences, our computational approach allowed to obtain data relevant for viral fusion in general. In fact, a major limitation for studying the structure of the post-fusion complex is that crystallized proteins including the stem region and the TM anchor could not be obtained [5-8]. Thus, the described interaction between the fusion and trans-membrane peptides, likely to occur in the post-fusion conformation, cannot be directly visualized in the crystal structure. This limitation gives to sequence based modeling efforts a crucial role in the sketching of a molecular interpretation of the fusion process, opening the way to rationally designed peptide-based antiviral therapy [25].

## Methods

### Protein sequence dataset

Twelve HCV protein sequences spanning the core (1–190 aa) and E1 (191–383 aa) regions of HCV were randomly selected from the HCV sequence database available at . Two sequences per each of the worldwide distributed 1a, 1b, 2a, 3a, 4a, 5a genotypes were downloaded for subsequent analysis. Accession numbers are: [GenBank:D10749, GenBank:AF011751] (genotype 1a); [GenBank:AB016785, GenBank:AB049087] (genotype 1b); [GenBank:AB047639, GenBank:AF169002] (genotype 2a); [GenBank:AF046866, GenBank:D17763] (genotype 3a); [GenBank:Y11604, GenBank:D45193] (genotype 4a); [GenBank:AF064490, GenBank:Y13184] (genotype 5a).

### Sequence homology analysis

The sequences were pairwise compared and their sequence homology was scored by means of ClustalW algorithm (BioEdit software). The between-genotype homology matrices relative to the fusion peptide (FP), the C-terminal domain (CT), and the Core fragment (Core), were then in turn pairwise compared by means of the Pearson correlation coefficient (*r*). The scoring of an elevated Pearson correlation between the homology matrices relative to two different peptides is widely recognized to be a signature of their functional and physical interaction [21].

### Recurrence Quantification Analysis

In order to complement the co-evolution approach and to derive a further proof of our hypothesis, we applied a Recurrence Quantification Analysis (RQA) on selected sequences by means of their Kyte-Doolitle hydrophobicity profile. This procedure is independent on protein homology and allows direct comparison of the two interacting proteins for the presence of common hydrophobicity patterns [30,32]. On this basis, the analyzed fragments were retrieved from one sequence per each genotype of the dataset. Sequence accession numbers were [GenBank:AF011751, GenBank:AB016785, GenBank:AB047639, GenBank:AF046866, GenBank:Y11604, GenBank:Y13184] for genotype 1a, 1b, 2a, 3a, 4a, 5a, respectively.

Basically the protein sequence is considered as an ordered series of numbers whose elements are the values of Kyte-Doolitle hydrophobicity of the residues along the sequence. In the case of cross-recurrence mode, two different sequences A and B are compared as for all the possible (a,b) ordered pairs of residues, whenever the i-th residue of protein A has a very similar hydrophobicity with residue j-th of protein B, a pixel is darkened in the matrix having as rows and columns the different residues of protein A and B respectively.

We used two numerical descriptors of cross-recurrence, the percentage of recurrent pairs, called REC (pairs of residues having very similar hydrophobicity in the two sequences/total number of distinct pairs) and the percentage of "deterministic recurrences", called DET (contiguous recurrent pairs/total recurrent pairs). The threshold for two i and j residues to be considered as very similar in hydrophobicity and consequently the ij pair to be recurrent was set to 5% of mean distance between residues (i.e. two residues are considered as recurrent if their difference in hydrophobicity is less than the 5% of mean distance in hydrophobicity across all the considered pairs). The above rules allow for the computation of all the amount of cross-recurrence (both as REC and DET) for all the possible peptide pairs. Where indicated we also used RQA descriptor called laminarity (LAM) that corresponds to the percentage of vertical (horizontal) recurrent lines in the cross recurrence plot [23].

## Abbreviations

HCV: Hepatitis C Virus; FP: Fusion Peptide; TM: Trans-membrane; CT: C-terminal; IT: Intervening tract; RQA: Recurrence Quantification Analysis; TBE: Tick Borne Encephalitis Virus; DET: percentage of deterministic recurrences; REC: percentage of recurrent pairs; LAM: laminarity i.e. percentage of recurrent lines in the cross recurrence plot.

## Authors' contributions

RB had been involved in conception and design of the study, sequence homology analysis, interpretation and drafting the manuscript; AC carried out sequence retrieving, sequence homology analysis and manuscript revision; ET contributed to sequence homology analysis; CM contributed to sequence homology analysis; MC contributed to sequence homology analysis and manuscript revision; MR contributed to revise the paper; GES contributed to revise the paper; AG participated in conception and design of the study, performed RQA and statistical analysis and critically revised the manuscript; ARC had been involved in conception and design of the study, data analysis, interpretation and drafting the manuscript. All the authors had given final approval of the version to be published.

## Supplementary Material

Additional file 1 **Supplementary Fig. 1**. Evidence for a correlation between REC and DET descriptors. Click here for file

Additional file 2 **Supplementary Table 1**. General dataset including computation of the first principal component (Factor1). Click here for file

Additional file 3 **Supplementary Table 2**. Analysis of Variance of the autologous/heterologous comparison. Click here for file

Additional file 4 **Supplementary Fig. 2**. FP-CT interaction as compared to NS4B-CT (upper panel) and Core-CT (lower panel) in the DET plane. The identity line allows to appreciate the differences in interaction strength. Click here for file

Additional file 5 **Supplementary Fig. 3**. E1TM-E2TM interaction as compared to NS4B-CT (upper panel) and Core-CT (lower panel) in the DET plane. The identity line allows to appreciate the differences in interaction strength. Click here for file

Additional file 6 **Supplementary Fig. 4**. E1TM-E2TM interaction as compared to FP-CT in both DET (upper panel) and REC (lower panel) planes. Click here for file

Additional file 7 **Supplementary Fig. 5**. Sequence order dependency of NS4B-CT, FP-CT and E1TM-E2TM interactions. The graph reports as black dots the REC and DET values of randomly shuffled pairs; arrows indicate the location of native interaction as well as the location of the mean of the random sample for the three NS4B-CT, FP-CT and E1TM-E2TM comparisons. Click here for file
