# Supplementary figures and images for: A computational approach identifies two regions of Hepatitis C Virus E1 protein as interacting domains involved in viral fusion process
Source: BMC Struct Biol. 2009 Jul 29;9:48. doi: 10.1186/1472-6807-9-48 (PMC2732612; doi:10.1186/1472-6807-9-48)

## Slide 1
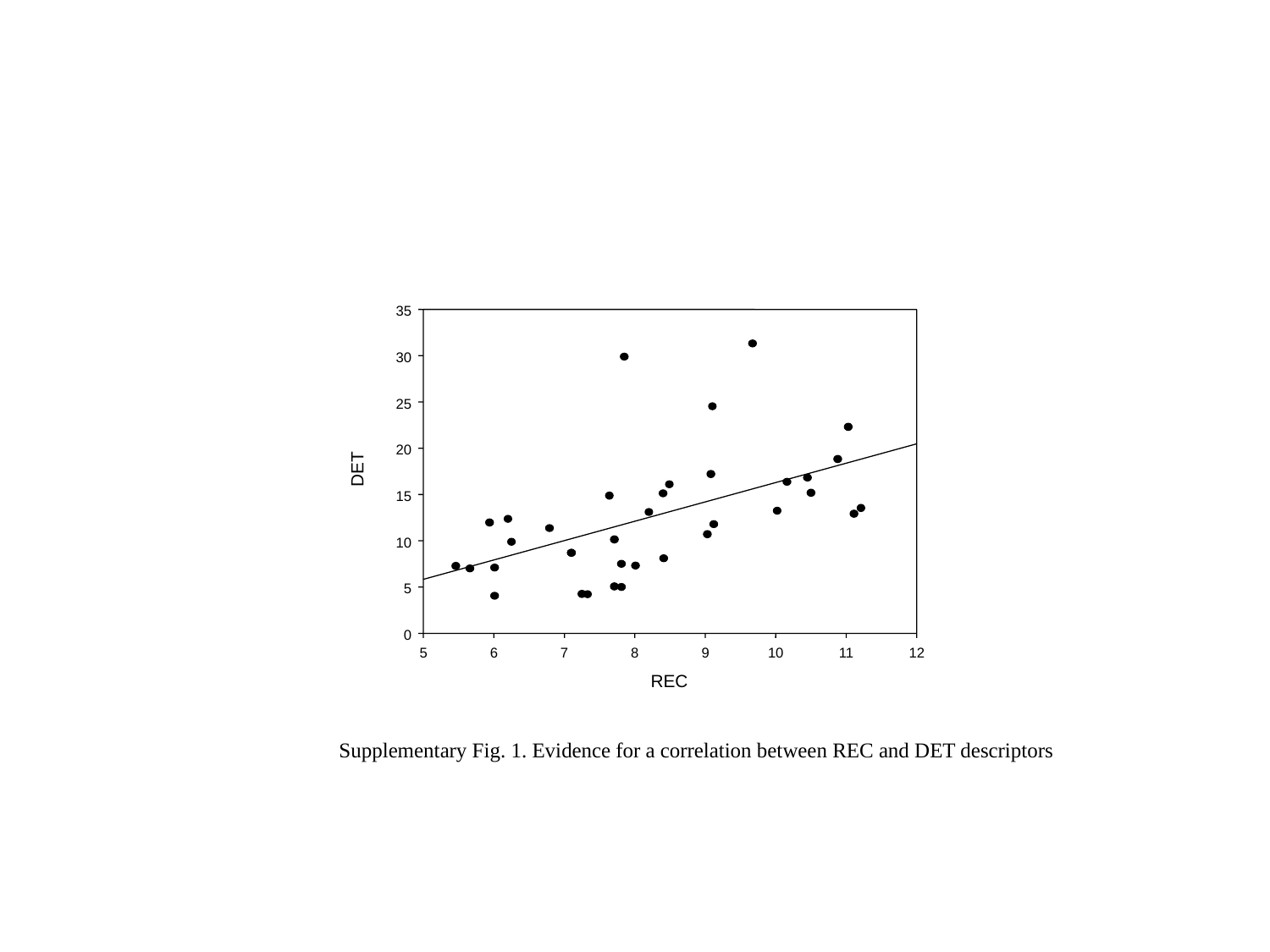

35
30
25
20
DET
15
10
5
0
5
6
7
8
9
10
11
12
REC
Supplementary Fig. 1. Evidence for a correlation between REC and DET descriptors

Supplement: Additional file 1 — Supplementary Fig. 1. Evidence for a correlation between REC and DET descriptors. [file 1472-6807-9-48-S1.ppt]

## Slide 1
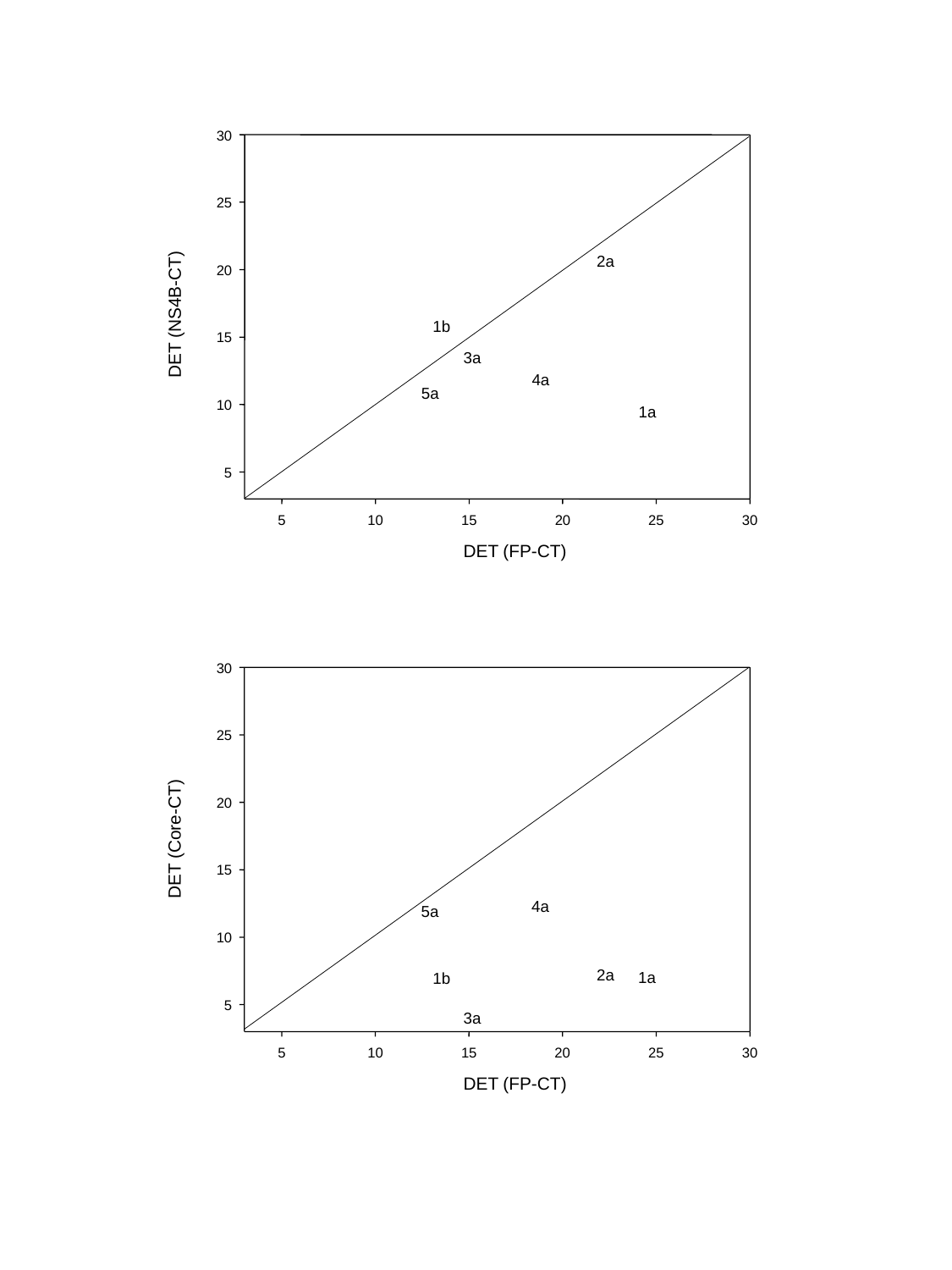

30
25
2a
20
DET (NS4B-CT)
1b
15
3a
4a
5a
10
1a
5
5
10
15
20
25
30
DET (FP-CT)
30
25
20
DET (Core-CT)
15
4a
5a
10
2a
1a
1b
5
3a
5
10
15
20
25
30
DET (FP-CT)

Supplement: Additional file 4 — Supplementary Fig. 2. FP-CT interaction as compared to NS4B-CT (upper panel) and Core-CT (lower panel) in the DET plane. The identity line allows to appreciate the differences in interaction strength. [file 1472-6807-9-48-S4.ppt]

## Slide 1
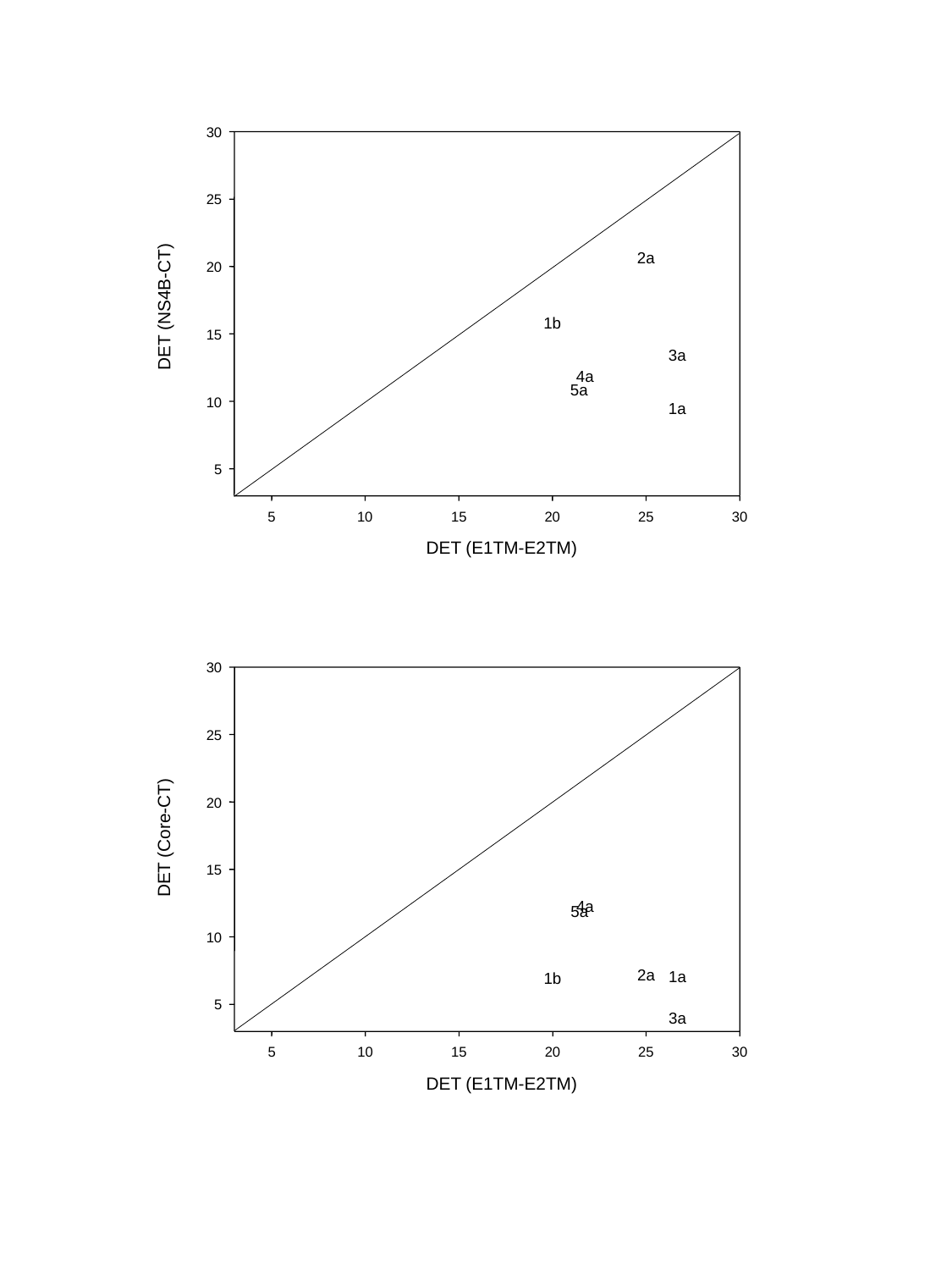

30
25
2a
20
DET (NS4B-CT)
1b
15
3a
4a
5a
10
1a
5
5
10
15
20
25
30
DET (E1TM-E2TM)
30
25
20
DET (Core-CT)
15
4a
5a
10
2a
1a
1b
5
3a
5
10
15
20
25
30
DET (E1TM-E2TM)

Supplement: Additional file 5 — Supplementary Fig. 3. E1TM-E2TM interaction as compared to NS4B-CT (upper panel) and Core-CT (lower panel) in the DET plane. The identity line allows to appreciate the differences in interaction strength. [file 1472-6807-9-48-S5.ppt]

## Slide 1
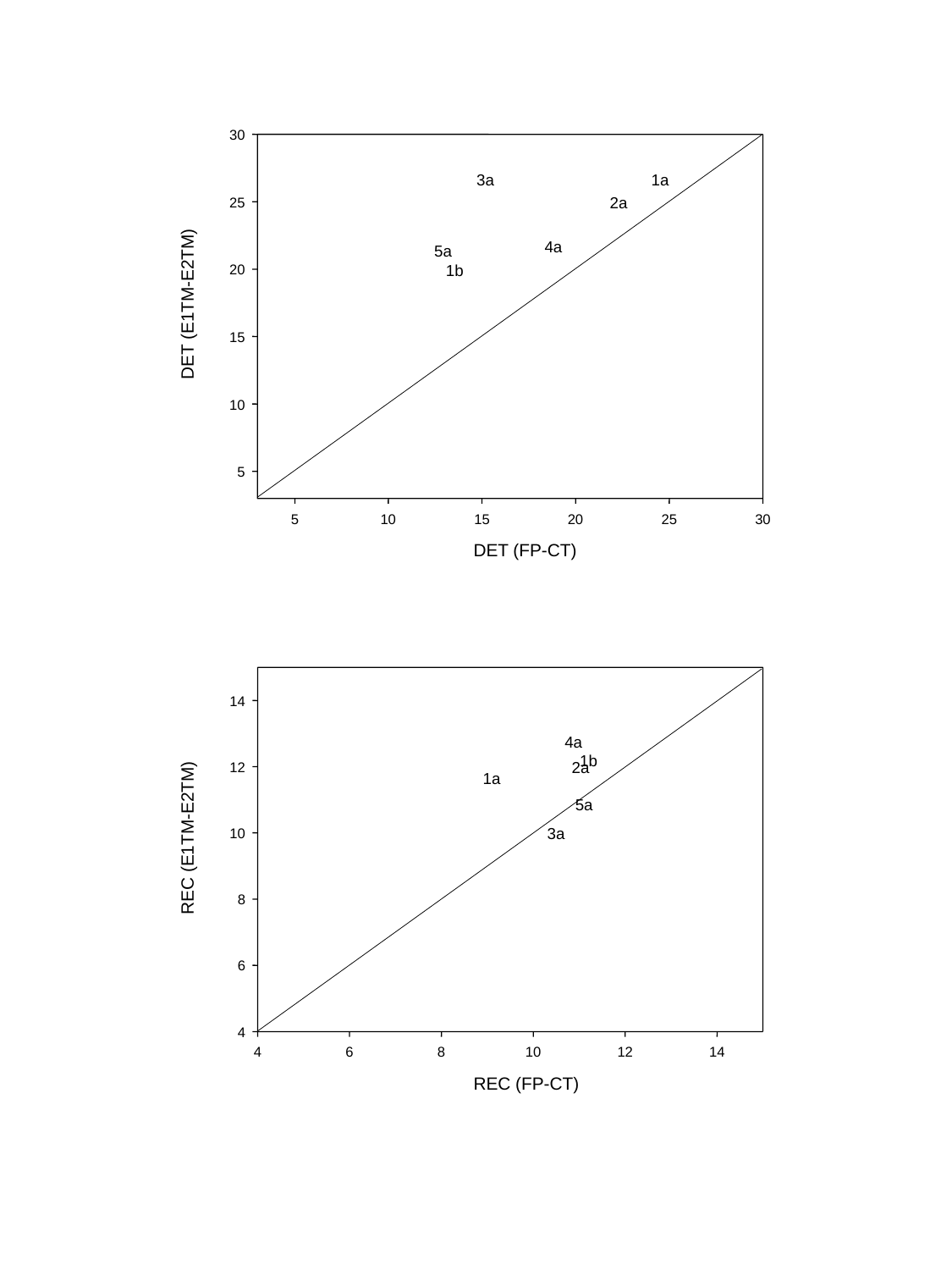

30
3a
1a
2a
25
4a
5a
1b
20
DET (E1TM-E2TM)
15
10
5
5
10
15
20
25
30
DET (FP-CT)
14
4a
1b
2a
12
1a
5a
3a
10
REC (E1TM-E2TM)
8
6
4
4
6
8
10
12
14
REC (FP-CT)

Supplement: Additional file 6 — Supplementary Fig. 4. E1TM-E2TM interaction as compared to FP-CT in both DET (upper panel) and REC (lower panel) planes. [file 1472-6807-9-48-S6.ppt]

## Slide 1
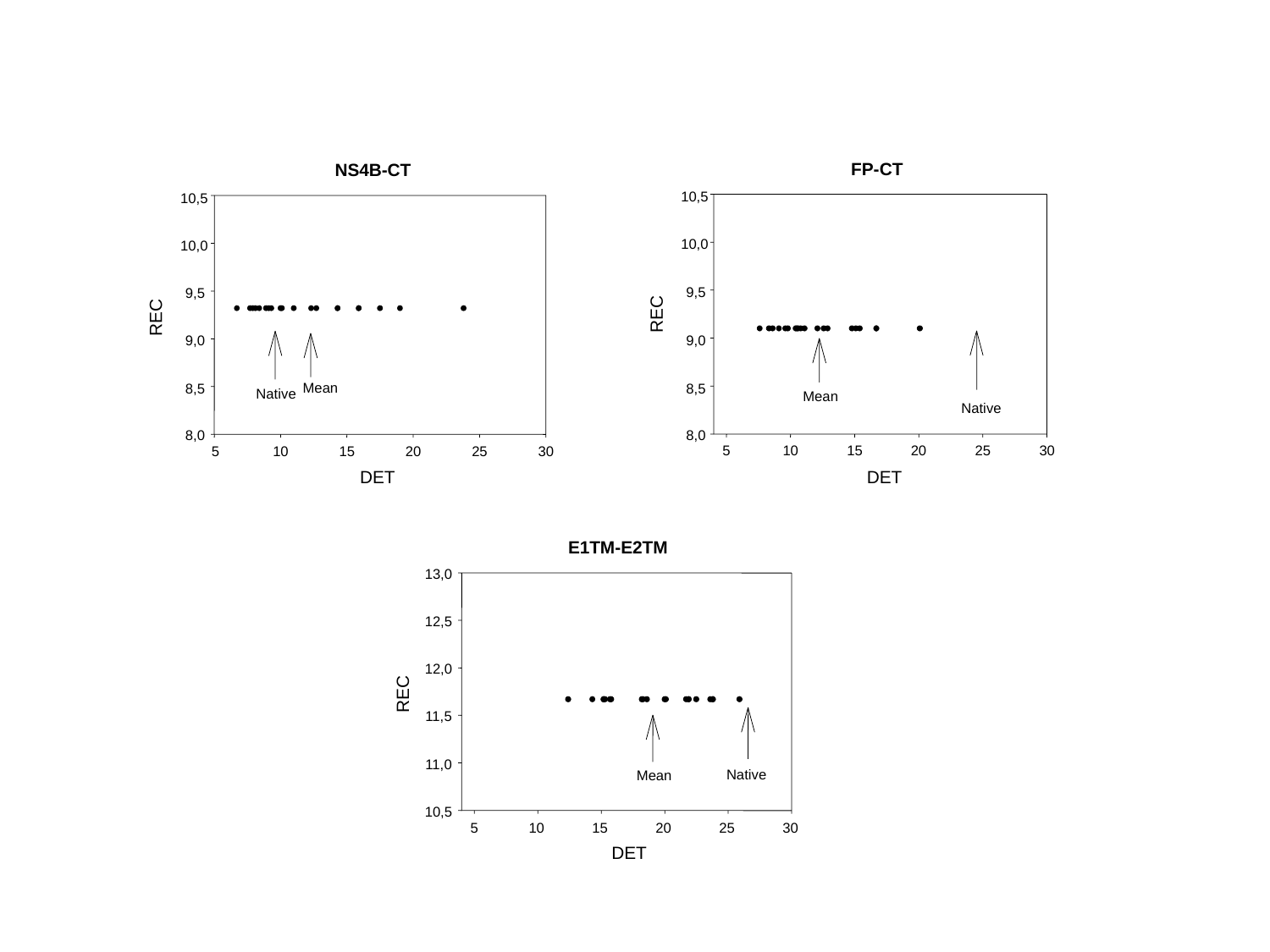

FP-CT
10,5
10,0
9,5
REC
9,0
8,5
Mean
Native
8,0
5
10
15
20
25
30
DET
NS4B-CT
10,5
10,0
9,5
REC
9,0
Mean
8,5
Native
8,0
5
10
15
20
25
30
DET
E1TM-E2TM
13,0
12,5
12,0
REC
11,5
11,0
Native
Mean
10,5
5
10
15
20
25
30
DET

Supplement: Additional file 7 — Supplementary Fig. 5. Sequence order dependency of NS4B-CT, FP-CT and E1TM-E2TM interactions. The graph reports as black dots the REC and DET values of randomly shuffled pairs; arrows indicate the location of native interaction as well as the location of the mean of the random sample for the three NS4B-CT, FP-CT and E1TM-E2TM comparisons. [file 1472-6807-9-48-S7.ppt]
