# Supplementary material for: A computational approach identifies two regions of Hepatitis C Virus E1 protein as interacting domains involved in viral fusion process
Source: BMC Struct Biol. 2009 Jul 29;9:48. doi: 10.1186/1472-6807-9-48 (PMC2732612; doi:10.1186/1472-6807-9-48)
Supplement: Additional file 2 — Supplementary Table 1. General dataset including computation of the first principal component (Factor1). [file 1472-6807-9-48-S2.doc]

| Supplementary Table 1  General dataset including computation of the first principal component (Factor1) | | | | | |
| --- | --- | --- | --- | --- | --- |
| Couple | REC | DET | Type | Genotype | Factor1 |
| Core-IT 1a | 8.20 | 13.09 | heterologous | 1a | -0.39082 |
| Core-IT 1b | 8.01 | 7.32 | heterologous | 1b | 2.09261 |
| Core-IT 2 | 7.71 | 5.06 | heterologous | 2a | 0.97423 |
| Core-IT 3 | 7.81 | 5.00 | heterologous | 3a | 1.11295 |
| Core-IT 4 | 8.40 | 15.12 | heterologous | 4a | 0.38636 |
| Core-IT 5 | 7.81 | 7.50 | heterologous | 5a | 0.68092 |
| Core-FP 1a | 7.10 | 8.70 | heterologous | 1a | 1.31625 |
| Core-FP 1b | 6.79 | 11.36 | heterologous | 1b | 1.08907 |
| Core-FP 2 | 6.25 | 9.88 | heterologous | 2a | 1.78022 |
| Core-FP 3 | 7.33 | 4.21 | heterologous | 3a | 0.98908 |
| Core-FP 4 | 7.25 | 4.25 | heterologous | 4a | 1.43052 |
| Core-FP 5 | 7.10 | 8.70 | heterologous | 5a | 1.00192 |
| Core-CT 1a | 6.01 | 7.10 | heterologous | 1a | -0.32771 |
| Core-CT 1b | 5.66 | 7.00 | heterologous | 1b | 0.22908 |
| Core-CT 2 | 5.46 | 7.28 | heterologous | 2a | 0.10582 |
| Core-CT 3 | 6.01 | 4.05 | heterologous | 3a | 0.65970 |
| Core-CT 4 | 6.20 | 12.36 | heterologous | 4a | -0.00755 |
| Core-CT 5 | 5.94 | 11.97 | heterologous | 5a | 1.35340 |
| FP-IT 1a | 7.71 | 10.13 | autologous | 1a | 0.02954 |
| FP-IT 1b | 9.67 | 31.31 | autologous | 1b | -0.53035 |
| FP-IT 2 | 10.16 | 16.35 | autologous | 2a | -0.82616 |
| FP-IT 3 | 10.45 | 16.82 | autologous | 3a | -0.79740 |
| FP-IT 4 | 8.49 | 16.10 | autologous | 4a | 0.27168 |
| FP-IT 5 | 9.08 | 17.20 | autologous | 5a | -0.58273 |
| FP-CT 1a | 9.10 | 24.52 | autologous | 1a | -0.72050 |
| FP-CT 1b | 11.21 | 13.54 | autologous | 1b | -0.59723 |
| FP-CT 2 | 11.03 | 22.30 | autologous | 2a | -0.90747 |
| FP-CT 3 | 10.50 | 15.18 | autologous | 3a | -1.02803 |
| FP-CT 4 | 10.88 | 18.82 | autologous | 4a | -1.05173 |
| FP-CT 5 | 11.11 | 12.92 | autologous | 5a | -0.72050 |
| CT-IT 1a | 8.41 | 8.10 | autologous | 1a | -1.22758 |
| CT-IT 1b | 9.12 | 11.78 | autologous | 1b | -1.35487 |
| CT-IT 2 | 9.03 | 10.70 | autologous | 2a | -1.39866 |
| CT-IT 3 | 10.02 | 13.24 | autologous | 3a | -1.48947 |
| CT-IT 4 | 7.64 | 14.87 | autologous | 4a | -0.71148 |
| CT-IT 5 | 7.85 | 29.89 | autologous | 5a | -0.83315 |
