# Supplementary material for: A computational approach identifies two regions of Hepatitis C Virus E1 protein as interacting domains involved in viral fusion process
Source: BMC Struct Biol. 2009 Jul 29;9:48. doi: 10.1186/1472-6807-9-48 (PMC2732612; doi:10.1186/1472-6807-9-48)
Supplement: Additional file 3 — Supplementary Table 2. Analysis of Variance of the autologous/heterologous comparison. [file 1472-6807-9-48-S3.doc]

| Supplementary Table 2.  Analysis of Variance of the autologous/heterologous comparison | | | | | | | | |  | |  | |  |
| --- | --- | --- | --- | --- | --- | --- | --- | --- | --- | --- | --- | --- | --- |
| Source | DF |  | Dependent variable | | | | | | | | | | |
|  |  |  | REC | |  | DET | |  | | Factor 1 | | | |
|  |  |  | F value | p |  | F value | p |  | | F value | | p | |
| type | 1 |  | 47.51 | <.0001 |  | 21.06 | 0.0001 |  | | 61.46 | | <.0001 | |
| geno | 5 |  | 0.46 | 0.8023 |  | 0.60 | 0.6997 |  | | 0.34 | | 0.8817 | |
| type*geno | 5 |  | 1.02 | 0.4278 |  | 0.33 | 0.8923 |  | | 1.04 | | 0.4160 | |
